# Joint Torque and Mechanical Power of Lower Extremity and Its Relevance to Hamstring Strain during Sprint Running

**DOI:** 10.1155/2017/8927415

**Published:** 2017-07-12

**Authors:** Yunjian Zhong, Weijie Fu, Shutao Wei, Qing Li, Yu Liu

**Affiliations:** ^1^College of Physical Education, Nanchang University, Nanchang 330031, China; ^2^School of Kinesiology, Shanghai University of Sport, Shanghai 200438, China; ^3^Key Laboratory of Exercise and Health Sciences of Ministry of Education, Shanghai University of Sport, Shanghai 200438, China; ^4^Teaching and Research Division of Physical Culture, Tsinghua University, Beijing 100083, China

## Abstract

The aim of this study was to quantify the contributions of lower extremity joint torques and the mechanical power of lower extremity muscle groups to further elucidate the loadings on hamstring and the mechanics of its injury. Eight national-level male sprinters performed maximum-velocity sprint running on a synthetic track. The 3D kinematic data and ground reaction force (GRF) were collected synchronously. Intersegmental dynamics approach was used to analyze the lower extremity joint torques and power changes in the lower extremity joint muscle groups. During sprinting, the GRF during the stance phase and the motion-dependent torques (MDT) during the swing phase had a major effect on the lower extremity movements and muscle groups. Specifically, during the stance phase, torque produced and work performed by the hip and knee muscles were generally used to counteract the GRF. During the swing phase, the role of the muscle torque changed to mainly counteract the effect of MDT to control the movement direction of the lower extremity. Meanwhile, during the initial stance and late swing phases, the passive torques, namely, the ground reaction torques and MDT produced by the GRF and the inertial movement of the segments of the lower extremity, applied greater stress to the hamstring muscles.

## 1. Introduction

Sprint running is a cyclical movement of alternate support and flight motions and combination of foot-strike and swing. The human body gains forward momentum by the strong push-off of the lower extremity during the stance phase [1, 2]. In terms of motion of each body segment, lower extremity motion is the key part of the entire sprint technique. The ability of the lower extremity muscle groups to perform specific work directly affects running speed and in turn interacts with the loading conditions of the muscle itself. This process may lead to muscle overload (e.g., hamstrings strain) [3, 4].

Several studies have used the inverse dynamics approach to quantify lower extremity joint torques during sprinting [5–8]. These findings are beneficial in examining the function of the lower extremity muscle groups during maximum-velocity sprint running and further determining muscle loading conditions. Specifically, during the stance phase, the large ground reaction force (GRF) generates contact torques simultaneously on lower extremity joints. Meanwhile, greater motion-dependent torques (MDT; e.g., inertia, Coriolis, and centrifugal forces) will be generated and acted upon each segment when lower extremity joints rapidly alternate between flexion and extension during the swing phase [9]. Torques generated by these external forces play a vital role in affecting the function of the lower extremity muscle groups during sprinting.

Currently, most studies on the different phases in sprinting and different levels of sprinters have focused on the GRF and lower extremity dynamics [10–14]. Analysis of the changes in joint muscle torques (MUS) covers several phases, including the stance phase of the second step after push-off [15], acceleration phase [6, 16], maximum-velocity phase [1, 8], and swing phase of the maximum-velocity [3]. The sprint technique varies greatly in the different phases and levels of sprinter subjects, resulting in much discrepancy on the characteristics of lower extremity joint MUS in these findings. Additionally, muscle power is an important biomechanical parameter in human gait analysis [17–19]. This analysis explains the formation of and control over human body segmental movements from energy and work generation perspectives. The results can be used to indirectly confirm the type of motion (concentric contraction or eccentric contraction) of the muscle groups (extensors or flexors) around joints. However, among these studies, only a few examined the muscle power of lower limbs [5, 16, 19], and the lower extremity joint torques and muscle power of a gait during maximum-velocity sprint running have not been analyzed yet.

On the other hand, hamstring strain injury is one of the most common injuries during sprinting [20, 21]. However, the underlying mechanisms of these injuries are still ambiguous, because most studies are based on the clinical muscle strain assumption [22]. Limited attempts have been made to measure ground reactions during overground sprinting, and few studies have used such data to estimate hamstring kinetics during stance and swing phases [23]. Therefore, understanding the coordination of muscular torque and the loading conditions of the hamstring during sprinting is beneficial to further quantify the torque component one by one during probing joint torques [24]. These parameters could be determined through an advanced inverse dynamics perspective, and the hamstring strain risk can be explored based on the insight mechanical mechanism. In this study, we adopted the intersegmental dynamics approach to break down the net joint torque (NET) of a gait during maximum-velocity sprint running into the MUS, MDT generated by movement, contact torques (EXF) generated by ground reaction force (GRF), and gravitational torques (GRA) [7, 25, 26].

Based on the above consideration, the purpose of this study was to quantify the contributions of lower extremity joint torques and the mechanical power of lower extremity muscle groups to further elucidate the loadings on hamstring and the mechanics of its injury. We hypothesized that the EXF and MDT could play an important role in the contributions of lower extremity joint torques during stance and swing phases, respectively. The effects of active and passive joint torque components on the risk of hamstring injury were also determined.

## 2. Methods

### 2.1. Subjects

Eight male national level sprinters (age: 21.1 ± 1.9 years, mass: 74.7 ± 4.1 kg, height: 181.5 ± 3.9 cm) were recruited to participate in this study. The best personal performance of the sprinters for 100 m ranged from 10.27 s to 10.80 s. These participants had no history of lower extremity injuries in the six months prior to the study. The study was approved by the local ethical committee. Each subject signed informed consent forms after all questions were answered satisfactorily.

### 2.2. Data Collection

The athletes performed maximal-effort sprints on a synthetic track, and three-dimensional (3D) kinematics data were obtained at a sampling rate of 300 Hz from eight Vicon high-resolution cameras (Vicon Motion Capture, Vicon, England). A total of 57 retroreflective markers (14.0 mm diameter) comprising the plug-in gait marker set used in our previous study [27] were attached to the lower limb to define hip, knee, and ankle joints. The calibration volume for the kinematics data collection was 10.0 × 2.5 × 2.0 m^3^ and centered 40 m from the sprint start line. A recessed Kistler force plate (60 × 90 cm^2^) (Kistler 9287B, Kistler Corporation, Switzerland) located at 40 m from the sprint start line was used to measure the GRF. The force signals were amplified and recorded in the Vicon system at a sampling rate of 1200 Hz. After a 5–10 min warm-up, each sprinter wearing track spikes performed three valid trials with sufficient rest intervals. The trial in which no markers dropped and either foot of the subject successfully hit the force plate was analyzed.

### 2.3. Data Analysis

Visual 3D (Version 3.390.23, C-Motion Corporation, USA) was used to calculate the kinematics and dynamics data. The tracks of the markers were filtered by Butterworth low-pass digital filter at a cutoff frequency of 17 Hz [28]. The GRF data were low-pass filtered with a cutoff frequency of 55 Hz.

The joint angles and angular velocity of lower extremity were also computed based on the visual 3D requirement for a skeletal framework. Meanwhile, the intersegmental dynamics model was used, in which the algorithm applied in our previous studies and other former studies was modified [23, 27]. Briefly, the analysis was conducted by a customized program based on intersegmental dynamics model and by inputting limb kinematics, anthropometric data, and GRF [27]. The lower extremity, that is, hip, knee, and ankle, was considered as a generalized linked-segment model. Based on free body diagrams of the segments, the dynamic formula of motion was derived by using the Newton-Euler equations applied to each segment. Torques at each joint can be separated into five categories, namely, NET, GRA, MDT, EXF (termed as ground reaction torques in this study), and MUS, with the first category being the sum of the rest:

(1) $$\begin{array}{c} NET=MUS+GRA+EXF+MDT. \end{array}$$

NET is the sum of all the torque components acting at a joint. MUS is mainly generated by muscle contractions. GRA results from gravitational forces acting at the center of mass of each segment. EXF is generated at joints by the GRF acting on the foot. MDT arises from the mechanical interactions occurring between limb segments and is the sum of all interaction torques produced by segment movements, such as angular velocity and angular acceleration of segments.

The muscle power of the lower extremity joint (*P_j_*) was calculated as follows [16]:

(2) $$\begin{array}{c} P_{j}=M_{j}\times \omega_{j}, \end{array}$$

where *M_j_* is the MUS generated by agonist muscles and antagonist muscles during joint movement and *ω_j_* is the angular velocity of the joint.

If the values of a joint torque and angular velocity were positive, the function of such torque was classified as extension joints (plantar flexion for ankle). By contrast, the function of torque was categorized as flexion joints (dorsiflexion for ankle). Positive muscle power indicated that the muscle torque and joint angular velocity were moving toward the same direction, and the muscle was doing concentric contraction. By contrast, negative muscle power indicated eccentric contraction of the muscle.

### 2.4. Statistics

Data were expressed as mean ± *SD*. One-way ANOVA and paired *t*-tests were used to determine the differences between joints and extensors/flexors in all joint kinematics and kinetic variables, respectively (10.0, SPSS Inc., Chicago, IL, USA). The significance level for all statistical tests was set at *α* = 0.05.

## 3. Results

**Figure 1:**
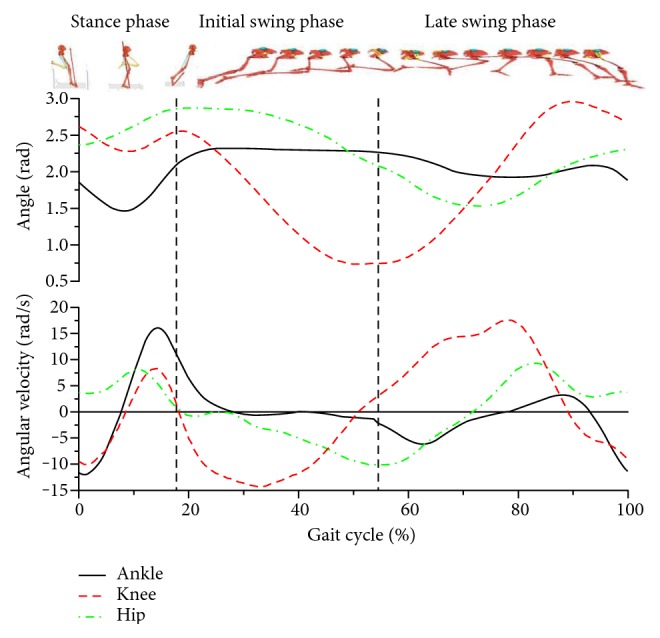
Changes in the lower extremity joint angles and angular velocity (rad/s) in a gait cycle. Values on the *x*-axis represent percentages of total gait time.

The mean sprinting speed during data collection for the eight subjects was 9.7 ± 0.3 m/s. A stride cycle in this study comprised two phases, namely, stance and swing phases. The stance phase was defined as the phase in which the left foot of an athlete was in contact with the ground (the critical point was standardized instant of 17.7% ± 1.2%) (Figure 1). The following swing motion of the left leg was defined as the swing phase. This phase was divided into two periods, namely, initial and late swing phases, based on the different timing. The demarcation timing was when the thigh reached an upright posture on the vertical line of the center of mass (standardized instant of 55.1% ± 2.3%).

Figure 1 and Table 1 show that the peak extension-flexion angular velocity in the ankle joint was significantly greater than that in the hip and knee joints. During the stance phase, the order of occurrence time of the peak values of joint extension was hip, knee, and ankle joints.

**Figure 2:**
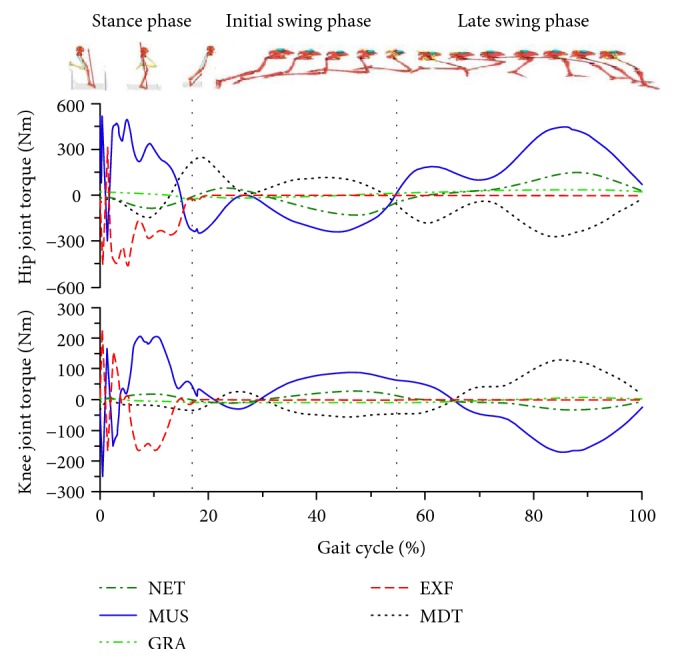
Torques of hip and knee joints in a gait cycle. Values on the *x*-axis represent percentages of total gait time. NET: NET joint torque; MUS: muscle torque; GRA: gravitational torque; EXF: contact torque; MDT: motion-dependent torque.

Figure 2 illustrates the torque components acting on the subjects' lower extremity segments. In terms of the intersegmental torque curves of hip, knee, and ankle joints during the maximum-velocity sprint running, during stance phase, the torques affecting the three lower extremity joints were mainly MUS and EXF (Figure 2, MUS, EXF, 0%–17%), while in the swing phase, the torques were primarily MUS and MDT (Figure 2, MUS, MDT, 17%–100%).

**Figure 3:**
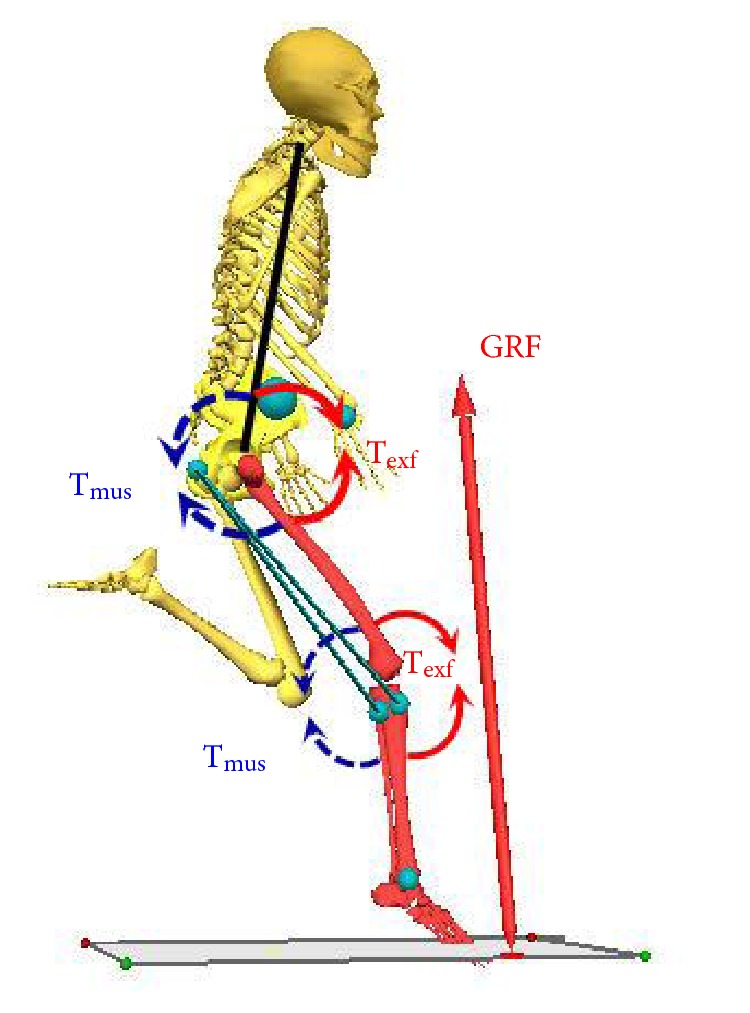
Force diagram of human body's initial contact with the ground during maximum-velocity sprint running. T_exf_: the torque generated at joints by the GRF acting on the foot; T_mus_: the torque generated by muscle contractions.

Figure 3 demonstrates that the GRF passed through the front of three joints which generated the hip flexor, knee extensor, and dorsiflexion torques.

**Figure 4:**
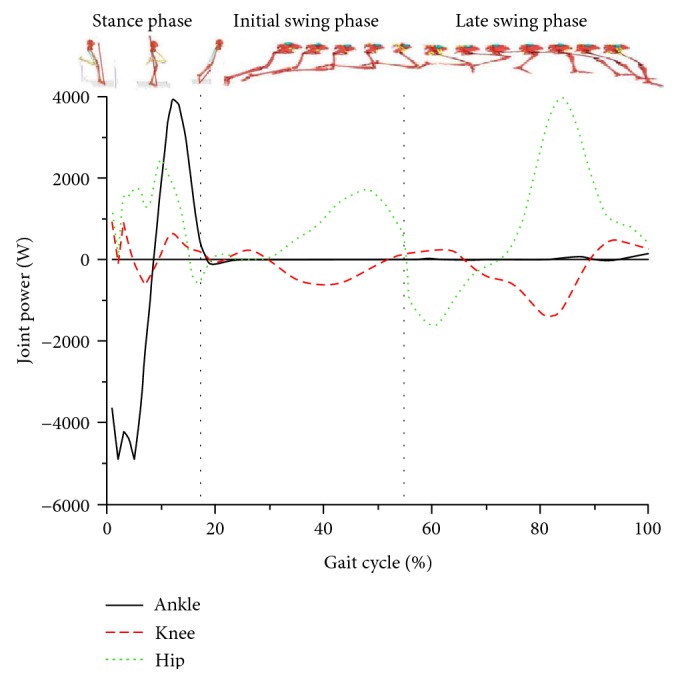
Muscle powers of the hip, knee, and ankle joints in a gait cycle.

Figure 4 illustrates that, in a gait of sprinting, the hip, knee, and ankle joint muscles all performed negative work with different extents. When these force-generated muscles performed negative work, the contraction type was eccentric and showed passive contraction. During the stance phase, the positive power of the ankle showed greater value compared with the power of the knee and hip joints.

Table 2 shows that the peak powers of the hip extensors, knee flexors, and ankle plantarflexors were all significantly greater than those of the hip flexors, knee extensors, and ankle dorsiflexors. Moreover, the peak power of the hip extensors was significantly greater during their performance of positive work than negative work. By contrast, the peak powers of the ankle plantarflexors and knee flexors were significantly greater during their performance of negative work than positive work.

## 4. Discussion

### 4.1. Stance Phase

Analysis of all lower extremity joint torques during the stance phase showed that the EXF, which was the ground reaction torque, acting on the hip joint was primarily manifested as hip flexor torque. The hip joint muscle torque was mainly manifested as hip extensor torque. Similarly, the torque generated by the EXF acting on the knee and ankle joints was the knee extensor and dorsiflexion torques. The MUS were mainly manifested as the knee flexor and plantar flexion torques. Thus, the hamstring muscles were under considerable demand, because they served both for knee flexors and hip extensors. Apparently, these muscles were vulnerable to strain during the stance phase of sprinting.

The hip joint continued to perform extension during the entire stance phase (Figure 1, hip). The GRF passed through in the front of the hip joint (Figure 3) during the initial ground contact and generated the EXF that caused the hip flexion (Figure 2, hip, EXF). Meanwhile, the hip extensors performed positive work and generated hip extensor torque (Figure 2, hip, MUS) with peak power as high as 1106 ± 231 W (Figure 4, hip). During this earlier stage, the peak hamstring force across the hip joint can be reasonably estimated based on the MUS values at the hip. Additionally, the hip extensor torque was more than the torque generated by the hip extensor group during the late swing and initial stance phases, but was also a torque buffering the EXF to make the hip joint continue to extend while preventing it from hyperflexion.

Along with the GRF passing through the back of hip joint, the hip extensor torque was produced when the hip flexors performed negative work and a peak muscle power appeared briefly. From this instant until the mid-stance phase, the hip extensors performed positive work, and muscle power was maintained at a greater level with peak power as high as 2658 ± 937 W during the mid-stance phase. The knee extensor torque performed positive work (Figure 4, knee) during the mid- and late stance phases (Figure 2, knee, MUS) to resist the knee flexor torque generated by the GRF to rapidly extend the knee joint with peak power of 981 ± 172 W.

A complex phenomenon occurred during the knee flexion period during the initial stance phase when the knee extensor twice reached its peak values that would have been observed while negative work was performed. This phenomenon would induce great loads to act on the hamstrings along with the loads generated by hip extensors as mentioned above. Meanwhile, the hamstrings also encountered the knee extensors to generate a large flexor torque (Figure 2, hip and knee, MUS), which further indicates a high strain injury risk exists with loadings induced by both active hip extension and knee flexion during the initial stance phase (Figure 3).

During the push-off, the MUS (knee extensor torque) and power values decreased rapidly and were maintained at low level (Figure 2, knee, MUS). The muscle power of the knee joint was also lower than that of the other joints, because of its lower angular velocity during the stance phase. The angular velocity of the knee joint was lower than that of the other joints as shown by the curves of joint angular velocity and muscle power during the entire stance phase. Therefore, the power value of knee joint muscle groups was lower during the entire stance phase and considerably lower than that of the hip and ankle joint muscle groups. This result coincides with the findings of Bezodis et al. [5]. We believed that the major function of knee joint was to maintain the height of the human body's center of mass and to deliver energy from the hip joint to the ankle joint. Although Johnson also drew a similar conclusion [16], the findings were relatively different. Johnson showed that the muscle power of the knee joint was quite low during the initial stance phase and reached greater power in the mid-stance phase with a peak of 1544 ± 512 W. However, muscle power continued to decline until the foot was off the ground. The discrepancies in the findings might be due to the distinct focus and the acceleration phase of the sprint in Johnson's study.

For the ankle joint torque, the major torque acting on ankle joint was the EXF and MUS, which existed primarily during the stance phase (Figure 2, ankle). As the GRF passed in front of the ankle joint throughout, the contact torque was dorsiflexion torque all along (Figure 2, ankle, EXF). Moreover, the ankle joint muscle group appeared merely as plantar flexion torque during the stance phase to resist the contact torque that made the ankle dorsiflexion. During the initial stance phase, the plantar flexors performed negative work to absorb the GRF and generated the energy for the plantar flexion of ankle with peak power of 4930 ± 933 W. When entering the mid-stance phase, the plantar flexors were transformed from eccentric contraction (doing negative work) into concentric contraction (doing positive work) and pushed the body into the swing phase. During this process, the ankle plantar flexors were experiencing the stretch-shortening cycle and stored great amount of elastic energy prior to shortening, which was advantageous in the driving force supply and power output during the strike-stretch phase [29].

During the stance phase, the major torques acting on lower extremity joint torques were the MUS and EXF. The function of the lower extremity muscle groups was mainly to resist the contact torques generated by the GRF around the joints and to output positive work for power supply to maintain the velocity during the maximum-velocity phase [29]. However, the loads on the hamstring muscles were considerable, because they served both roles of the knee flexors and the hip extensors to counteract this effect of GRF. Furthermore, the anatomical structure of skeletal muscle linking the single joint to double-jointed joints of the lower limbs was advantageous in transmitting muscle power from the big joints to small joints (from proximal ends to distal ends). When viewing the peak angular velocity and peak muscle power of each joint during the stance phase sprinting, the peak angular velocity and peak extensor positive power appeared in turn from the proximal to distal joints. Meanwhile, during the stance phase, the peak angular velocity and peak muscle power of the ankle were significantly greater than those of the other joints. This result demonstrates that the elite athletes well delivered the hip joint muscle energy to the ankle joint to increase the capability of the ankle joint acting to the ground and better maintain velocity.

### 4.2. Swing Phase

During the swing phase, the primary hip joint MUS were in sequence as the hip flexor and hip extensor torques, while the dominant knee joint MUS manifested in sequence as the knee extensor and flexor torques. In terms of the sprint swing skills with the hip joint center served as an axis, the key of the swing velocity was the rapid flexion of the knee after toe-off as well as the COM acceleration and angular acceleration of the swing leg. Previous studies demonstrated that the muscle groups affecting the folding angle of the thigh and shank (the knee flexion) were the joint muscles, such as the biceps femoris long head, semitendinosus muscle, and semimembranosus, which were responsible for the dual duties of hip extension and knee flexion. When folding started in the initial swing phase, the hip joint was still at an extended status such that these joint muscles were kept at a greater level of activation, resulting in an active insufficiency of knee flexion [1]. Through quantifying the MUS and MDT, the knee flexor torque (Figure 2, knee, MDT) was found to be the major MDT acting on the knee joint during the initial swing phase. The MDTs, not the knee flexors (hamstrings), mainly contributed energy to make the shank fold. The primary MUS was the muscle extensor torque that performed negative work (Figure 4, knee) to control the movements and decelerate the knee flexion motion.

The MUS was manifested as the hip extensor torque which tensed the hamstring almost throughout the entire late swing phase to resist the MDT of the hip flexion (Figure 2, Hip, MDT), decelerate the hip joint flexion, and enter the next step of rapid pressing (hip extension) of the swing leg (Figure 2, hip, MUS). The hip extensors are shown in Figure 4 (hip) as a sequence of the antagonist (eccentric contraction) and agonist (concentric contraction) that performed negative and positive works, respectively, for the hip movements. The hip extensors, mainly for hamstring muscles, during the late swing phase showed peak positive and negative powers at 3996 ± 1120 and −1606 ± 781 W, respectively. Meanwhile, the knee flexors, including hamstring muscles, mainly manifested as muscle flexor torques in resisting the MDT by knee extension, and these muscles were performing negative work at this moment to decelerate the knee extension movement and transit to the stance phase. At this point, the knee joint muscle groups reached their peak power at −2104 ± 572 W. Therefore, the late swing phase of a sprint gait appeared to be the risk period when a hamstring strain would easily occur. These findings were in accordance with the previous studies of Thelen et al. [25], Chumanov et al. [30], and Yu et al. [31].

The major joint torques acting on the lower extremity joints during the swing phase were mainly the MUS and MDT. The inertial torque was the key factor in affecting the MUS, which was a major driving force for the movements of the thigh and shank. Under many circumstances, the MUS performed negative work to control movements. The effects of the ankle joint torques and muscle power on the lower extremity movements are quite limited because they are remarkably low during the swing phase.

## 5. Conclusion

The external and motion-dependent forces (e.g., inertial forces, Coriolis force, and concentric force) that acted on each segment of the human body had vital effects on the function of joint muscle groups during sprinting. During the stance phase, torque produced and work performed by the hip and knee muscles were generally used to counteract GRF. During the swing phase, the role of MUS changed to mainly counteract the effect of MDT to control the movement direction of the lower extremity. Meanwhile, during the initial stance and late swing phases, the passive torques, that is, EXF and MDT produced by GRF and the inertial movement of the segments of the lower extremity, applied greater stress to the hamstring muscles, which put these muscles at a higher risk of strains.

## Figures and Tables

**Table 1:** Comparison of peak joint extension-flexion angular velocity (*v*_PA_) between three joints during a gait circle.

	Hip joint		Knee joint		Ankle joint	
	Extension	Flexion	Extension	Flexion	Plantarflexion	Dorsiflexion
*v*_PA_ (rad/s)	9.32 ± *1.15*^∗^	9.95 ± *1.02*^†^	11.64 ± *1.11*^∗^	9.57 ± *0.95*^†^	16.20 ± *1.97*	11.51 ± *0.96*

^∗^^,†^Significantly different from ankle plantarflexion and dorsiflexion, respectively.

**Table 2:** Comparison of peak muscle power in the lower extremity muscle groups during a gait circle.

	Hip joint muscle		Knee joint muscle		Ankle joint muscle	
	Extensors	Flexors	Extensors	Flexors	Plantarflexors	Dorsiflexors
Peak positive power (W)	3996 ± *1120*^∗^^†^	1735 ± *339*	627 ± *113*^∗^	1010 ± *208*^†^	3954 ± *673*^∗^^†^	135 ± *49*
Peak negative power (W)	−1606 ± *781*^∗^	−630 ± *108*	−655 ± *126*^∗^	−1402 ± *372*	−4930 ± *933*^∗^	−96 ± *25*

^∗^Significantly different from the flexors of identical joint; ^†^significantly different from the peak negative power of identical joint muscle (the absolute value).
